# Supplementary material for: Are carbon emissions trading and green financial instruments synergistic? -Comprehensive quantitative research based on content analysis
Source: PLoS One. 2024 Mar 7;19(3):e0298601. doi: 10.1371/journal.pone.0298601 (PMC10919601; doi:10.1371/journal.pone.0298601)
Supplement: S1 Data — (ZIP) [file pone.0298601.s001.zip › Data/地级市Z_F工作报告.zip!/╔╜╬≈/╦╖╓▌╩╨/└┤╫╘╣1⁄2╓┌║┼í╛┬φ┐╦╩2╛▌═°í┐_2019.docx]

各位代表：

　　现在，我代表市人民政府向大会报告工作，请予审议，并请市政协委员和其他列席人员提出意见。

　　一、2018年工作回顾

　　2018年是全面贯彻党的十九大精神的开局之年。一年来，面对深刻变化的外部环境，在市委的坚强领导下，我们坚持以习近平新时代中国特色社会主义思想为指导，全面贯彻党的十九大和十九届二中、三中全会精神，深入贯彻习近平总书记视察山西重要讲话精神，认真落实省委、省政府“三大目标”定位要求，弘扬右玉精神，坚持新发展理念，落实“生态立市、稳煤促新”战略要求，统筹做好稳增长、促改革、调结构、惠民生、防风险各项工作，全市呈现经济平稳向好、社会和谐稳定的良好局面。

　　2018年，全市地区生产总值完成1065.6亿元，增长2.7%;固定资产投资完成248.1亿元，增长15.8%;一般公共预算收入完成90.4亿元，增长23.5%;社会消费品零售总额完成338.1亿元，增长8.5%;城镇居民人均可支配收入达到32849元，增长6%;农村居民人均可支配收入达到13423元，增长9.1%。城镇登记失业率2.5%，居民消费价格涨幅1.4%。

　　一年来，我们着力巩固提升传统产业，煤电行业发挥了经济发展“压舱石”作用。煤炭先进产能矿井达到32座，占比达到73%，超全省平均水平16个百分点，生产原煤1.5亿吨。电力运营装机规模达到1073万千瓦，新增装机规模45.86万千瓦;发电378.1亿度，增长15%。煤电行业对财政的贡献率达到51.54%。

　　一年来，我们扎实开展“转型项目建设年”活动，“2+7”产业格局更加清晰。我们既“稳煤”，又“促新”。围绕高端陶瓷和新材料、新能源、现代煤化工、生物医药、文化旅游、草牧业与农产品深加工、商贸物流等七大产业，谋划实施了一批科技含量高、转型效果好、就业岗位多的项目。通过投资项目承诺制改革、并联审批、市领导包联等措施，开复工项目390个，转型项目222个，完成投资152.6亿元，占固定资产投资的61.5%。转型发展效果显现，煤炭工业增加值占比降低6.2个百分点，超省定目标4.8个百分点;非煤产业增加值增长6.3%，超出规上工业10.8个百分点;战略性新兴产业增加值增长12%，高技术产业增加值增长21.4%。

　　一年来，我们不断深化改革开放，重点领域改革取得新进展。开发区改革创新方面，平鲁区、山阴县、应县开发区获批，全市开发区达到6个。“三化三制”改革扎实推进。朔州经济开发区迎宾大道和起步区开工建设。电力体制改革方面，新增电力直接交易用户7家，获批售电公司7家，完成交易29.84亿度，为用电企业降低成本1亿多元。组织风电企业与6个开发区签订年度长协交易电量5亿度，可惠及82家企业，全省首创。国资国企改革方面，“三供一业”分离移交完成年度任务。农村改革方面，应县全面完成农村集体产权制度试点改革。右玉县闲置凋敝宅基地整治盘活利用试点工作扎实推进。市、县、乡三级农村产权交易体系初步形成。金融改革方面，组建了朔州农商行和怀仁农商行，启动了右玉农信社改革。社会融资总额完成178亿元，是上年的2.6倍。科技创新方面，在固废利用、煤化工领域实施两项省科技重大专项，新认定高新技术企业10家，晋坤公司被认定为省级煤系高岭土工程技术研究中心。

　　财税改革方面，争取债券和转移支付72.6亿元，实施了10个PPP项目，市城投公司发行企业债18亿元，保障了基本运转、民生支出和基础设施建设。朔州经济开发区两个园区为入园企业降低土地使用成本。积极争取省财政支持，大秦公司变更所得税分享机制。落实费改税政策，征收水资源税和环境保护税5.8亿元。文化旅游发展方面，组建了市政银文化旅游产业投资公司。累计接待游客2913万人次。旅游综合收入达到261.24亿元，增长28.1%。民营经济发展方面，培养“小升规”企业57家。出台支持民营经济发展48条措施。招商引资方面，举办塞上长城国际旅游节、陶瓷交易会、羔羊肉交易会、亚洲粉煤灰大会“一节三会”。开展招商引资活动21次，签约项目196个，总投资699亿元。怀仁撤县设市，实现了我市行政区划调整的重大突破。

　　一年来，我们坚持补短板强弱项，推动全局性、基础性、长远性的重大事项实现新突破。历经11年建设，准朔铁路建成通车。历时9年，朔州机场立项即将获得国务院、中央军委批复。搁置5年的朔神高速重新落地。我们盯紧咬死、积极跟进，注册资本60亿元的苏晋能源公司最终回落朔州。由平鲁区、中煤集团、河北港务集团、太原铁路局合作建设的内陆港项目落地运营。起底排查项目注册地，引回部分风电企业注册朔州，确保税源不流失。特别是我们多次赴省进京、积极争取，骆惠宁书记、楼阳生省长、林武副书记等省领导亲自协调，全市人民期盼已久的大原高铁项目重新启动。

　　一年来，我们积极推进基础设施建设，城市功能更趋完善。完成投资32.93亿元，新改建城市道路76公里、各类管网227公里;恢河大桥通车，市区建成振华街桥、田家窑桥和三座人行天桥。改造50个公交候车亭，新增4条公交线路、70台纯电动公交车。棚户区改造开工4376套，基本建成4957套。城市建成区绿地率达到38.5%，绿化覆盖率达到42.87%。

　　一年来，我们不断强化县域经济发展，县(市、区)工作亮点纷呈、各具特色。怀仁市招商引资、固定资产投资、企业“小升规”均占到全市三分之一强，“五村联创”走在全市前列，玉龙化工园区以企招商成为我市园区建设的示范。朔城区南山生态治理成为生态建设的新标杆，神头海玫瑰庄园打造百万玫瑰花海小镇，推动旅游与农业深度融合。平鲁区大力推进内陆港建设，运营、在建电力总装机830万千瓦，成为全市综合能源基地建设的排头兵。山阴县被命名为“山西富硒谷子强县”，玉竹活性硅酸钙项目成为全市新材料行业中的亮点。应县农村集体产权制度改革走先一步，经纬通无车承运物流平台开启了我市物流业发展的新模式。右玉县玉龙生态观光牧场，成为文化旅游产业发展的引领项目。

　　一年来，我们大力实施乡村振兴战略，“三农”工作扎实推进。编制完成乡村振兴总体规划和“5+1”专项规划。发展特色现代农业。设立1亿元的农牧交错带发展基金。新增苜蓿种植面积5万亩，饲草料种植面积达到182万亩。粮食总产量28.3亿斤。肉羊饲养量610万只，奶牛存栏18.4万头。认定绿色农产品13个，有7个农产品荣获山西名牌产品，我市被命名为“全国杂粮强市”。提升农业产业化水平。农业产业化经营组织发展到1218个。应县南河种等4个市场列入农业部定点批发市场。古城乳业、龙首山粮油、金沙滩羔羊肉成为国家级龙头企业。全市农产品销售收入完成219.3亿元，增长6.73%。推进美丽乡村建设。投资4.7亿元，对270个村的环境集中整治。完成“四好农村路”、古长城旅游公路634公里。朔城区青钟村被评为“2018年全国生态文化村”。省级美丽宜居示范村、省级人居环境示范村达到42个，全省休闲农业与乡村旅游示范点达到12个。

　　一年来，我们全力打好三大攻坚战，风险防控、精准脱贫、污染防治取得明显成效。风险防控有力有效，投资近1.8亿元，解决了一批久拖不决的历史遗留信访问题。政府债务风险和金融风险总体可控。脱贫攻坚成效显著，投入资金3.1亿元，右玉县和平鲁区、山阴县脱贫摘帽，全市实现整体脱贫;全力攻坚深度贫困，3个贫困村、4654名贫困人口脱贫，脱贫攻坚连战连胜。生态环境持续改善，启动全域绿化，完成营造林33.48万亩。开展桑干河清河行动，“浚河、控污、固堤”效果初步显现。推动环保督察交办和反馈问题整改落实，问责64人。环境空气质量综合指数5.62，全省排名第2;达标天数237天，全省排名第3;未发生重污染天气。

　　一年来，我们致力民生改善，人民群众获得感幸福感安全感进一步增强。民生支出136.1亿元，占一般公共预算支出78.7%。省定六件实事全部完成。教育事业方面，省定国家义务教育基本均衡目标提前一年完成。市一中新校区等10所学校开工建设。卫生健康事业方面，公立医院改革进一步深化。各县(市、区)医疗集团组建完成、顺利运行。朔州大医院开工建设。文化体育事业方面，《右玉和她的县委书记们》在央视一套黄金时段播出，引起强烈反响。组织各类群众性文化体育活动90多项。送戏下乡731场。成功申办第十六届省运会。就业创业方面，城镇新增就业2.5万人，农村劳动力转移就业2.1万人，创业带动就业4500人，全部超额完成省定目标。社会保障方面，养老保险参保人数118.67万人，医疗保险参保人数138.94万人，基本实现应保尽保。为800名贫困大学生每人发放助学金2000元。安全生产方面，全市未发生较大及以上生产安全事故。社会综合治理方面，打掉涉黑涉恶团伙70个，破获各类案件445起，社会保持和谐稳定。

　　一年来，我们不断加强政府自身建设，营商环境进一步优化。坚持把学习习近平总书记重要讲话精神作为政府常务会议第一议题，用最新理论创新成果武装头脑、指导实践。强化“三基建设”，对标先进，完善首办责任制、服务承诺制、限时办结制和“13710”电子督办制，推动政府工作标准化规范化精细化管理。持续推进“放管服效”改革，取消行政许可事项97项，179项行政审批事项进入政务服务平台，一个窗口受理、一条龙服务、一站式办结。启动“12345”便民服务平台，办理群众诉求2万余条。落实全面从严治党要求，加强政府党风廉政建设，深入开展“力戒形式主义、狠抓工作落实”作风整顿，落实“六个决不允许”要求，干部作风明显好转，工作效率明显提升。自觉接受人大、政协监督，支持各民主党派、工商联、无党派人士参政议政，办理人大代表建议124件、政协委员提案142件。

　　认真开展民兵基地化训练，国防后备力量建设进一步加强，双拥工作扎实开展。妇女、儿童、老龄、残疾人、信访、宗教、人防、地震、应急、气象、档案、地方志、红十字会等各项事业都取得新成绩。

　　各位代表，过去一年取得的成绩来之不易。这是全市上下深入贯彻习近平新时代中国特色社会主义思想和党的十九大精神的结果，是省委、省政府和市委正确领导的结果，是市人大、市政协有效监督、全力支持的结果，是全市人民齐心协力、共同奋斗的结果。在这里，我代表市人民政府，向全市人民和所有关心支持朔州发展的各界人士，表示衷心的感谢和崇高的敬意!

　　与此同时，我们也清醒地看到，全市经济社会发展还存在不少困难和问题。主要是，煤炭产业发展方式粗放，产业链不完整，现代煤化工产业发展、大型煤炭物流基地建设进展缓慢;新兴产业发展不足、体量不大;实体经济面临不少困难，融资难融资贵问题突出，科技创新、人才支撑不足，支持实体经济发展的政策措施、营商环境有待进一步优化;交通基础设施建设滞后，严重制约高质量发展;大气、水、土壤污染治理任务艰巨;优质教育、医疗、养老服务等方面的供给与人民群众期盼有较大差距，城乡居民收入水平还不高，城乡区域协调发展水平需进一步提升;政府性债务、安全生产等领域的风险隐患不容忽视;政府职能转变还不到位，改革精神不足，创新意识不强，市场理念不浓，对经济运行的调度、管理、服务不够。我们一定要坚持底线思维，增强忧患意识，下大力气抓发展、补短板、强弱项、解难题，全力以赴做好政府工作!

　　二、2019 年工作安排

　　2019年是中华人民共和国成立70周年，是全面建成小康社会关键之年，是朔州建市30周年。政府工作的总体要求是：以习近平新时代中国特色社会主义思想为指导，认真学习贯彻党的十九大和十九届二中、三中全会精神，深入贯彻落实习近平总书记视察山西重要讲话精神和对右玉精神的重要指示精神，按照省委十一届七次全会和市委六届六次、七次、八次全会及市委经济工作会议总体部署，坚持稳中求进工作总基调，坚持新发展理念，坚持高质量发展，坚持“供改”与“综改”相结合，坚持深化市场化改革、扩大高水平开放，推进“生态立市、稳煤促新”战略，坚定不移推动转型发展，统筹做好稳增长、促改革、调结构、惠民生、防风险各项工作，继续打好“三大攻坚战”，保持经济持续健康发展和社会大局和谐稳定，奋力建设塞上绿洲、美丽朔州，努力提高人民群众获得感、幸福感、安全感。

　　主要预期目标是：全市地区生产总值同比增长7%左右，规模以上工业增加值增长8%，全社会固定资产投资增长10%，社会消费品零售总额增长7%左右，公共财政预算收入增长6.6%，城乡居民人均可支配收入分别增长7%左右和7%以上，城镇新增就业人数2.3万人，城镇登记失业率控制在4.2%以内。其他约束性指标完成省定任务。

　　实现上述目标，必须有效应对复杂多变的外部环境，努力把握国家宏观政策、结构性政策、社会政策等重大政策取向，落实“巩固、增强、提升、畅通”八字要求，聚焦主要矛盾，突出抓好事关全局和长远的重点工作，确保经济运行在合理区间。

　　重点抓好以下九项工作：

　　(一)全力推进项目建设，夯实转型发展基础

　　深化转型项目建设年活动，一季度为项目审批季，二季度为项目开工季，在基础设施、产业发展、社会民生、生态治理等领域，实施一批打基础、利长远、补短板、增动能的大项目、好项目，计划实施投资500万元以上项目475个，总投资1975亿元，年度计划投资377.7亿元;其中亿元以上项目189个，总投资1878亿元，年度计划投资324亿元。

　　基础设施建设突出“交通强市”。推进总投资590.9亿元的115个项目建设，打通交通瓶颈，加快构建立体综合交通运输体系。大原高铁总投资171亿元，要积极赴省进京汇报沟通，加快项目前期，3月底完成征拆，力争年内开工。改造韩原线及沿线车站，为“五一”前开通太原南到怀仁东动车组做好准备;加快总投资20.8亿元的朔山联络线建设。朔州机场总投资10亿元，年内开工。开通右平高速。开工建设总投资21亿元的朔神高速;启动市区东北环、应繁高速公路前期工作。开工建设总投资45亿元的阳方口—平鲁、东榆林—应县两条干线公路改扩建项目。完成总投资5.4亿元、总里程109公里的古长城旅游公路建设，完成总投资4.2亿元、总里程400公里的“四好农村路”建设。

　　各位代表，区位不优、交通不畅，是我市经济社会发展的最大短板。交通兴，则经济兴、百业兴。我们要把发展交通作为重大战略，举全市之力，建设“交通强市”，打造晋西北枢纽城市，强力推动大原高铁及其他铁路专线建设;把朔州机场打造成新的对外开放门户;用3—5年时间，打通南到宁武神池、北到怀仁右玉、东到山阴应县、西到平鲁偏关的快速通道，进而南下中原、北上蒙俄、东承京津、西接丝路，推动“铁公机”有机衔接，实现对内大畅通、对外大开放的立体综合交通格局，加快要素流动，促进区域协同，为未来发展蓄势储能!

　　产业发展突出重大项目落地。抓好总投资1188.6亿元的235个项目建设，推动新旧动能转换，加快“产业兴市”。推动煤炭物流、煤电、煤化工产业项目建设。推进总投资200亿元的平鲁内陆港项目。筹划朔城、山阴、怀仁内陆港项目。推动中电国际2×100万千瓦、平朔木瓜界2×66万千瓦、安太堡2×35万千瓦、同煤朔南2×35万千瓦低热值煤发电项目移出缓建停建名单。开工松蓝化工60万吨/年煤制乙二醇、新建能源公司300万吨/年低阶煤利用项目。开展平朔60万吨/年煤制乙二醇、75万吨/年煤制烯烃项目前期。推动各类优势产业项目建设。加快亿家亲3000万件/年日用瓷、华玺日用瓷自动化生产线等项目建设;加快三元炭素10万吨/年锂电子负极材料、玉竹60万吨/年石灰生产线、超牌20万吨/年煅烧高岭土等28个新材料项目建设;开工联盛15万千瓦、平朔10万千瓦等25个总装机169万千瓦的新能源项目;推进玉龙化工园区生物转化制备、8000吨医药中间体等6个生物医药项目建设;开工怀仁鲁沟生态旅游等27个农业产业化项目;推进罗克森300台/年大型发动机、天玛电液控制等装备制造项目建设;开工怀仁开发区标准化厂房项目。年内建成三通亿达内燃机起动机、晋能牛家岭风电等50个项目。

　　民生改善突出公共服务提升。推进总投资121亿元的66个项目建设。建成总投资19.8亿元的市一中等10所中小学校，建成总投资7.7亿元的怀仁陶瓷学院;加快总投资14.9亿元的朔州大医院项目建设;开工总投资6亿元的平朔生活区“双修”、总投资6.8亿元的奥体中心、总投资3.7亿元的右玉干部学院二期、总投资3.6亿元的朔城区一中改扩建等项目。

　　生态治理突出植绿兴水。推进总投资74.9亿元的59个项目建设，打造“朔州绿”。加快总投资12.16亿元的洪涛山生态恢复、朔城区南山生态治理等14个造林项目建设;推进总投资26.6亿元的桑干河综合治理和生态修复。

　　(二)全力构建“2+7+N”现代产业体系，推动经济高质量发展

　　围绕“稳煤”，巩固提升传统产业。煤电产业要在提质增效上下功夫，使内力更强、后劲更足、贡献更大。“减”“优”“绿”推进煤矿减量置换和减量重组，建设现代化矿井，提高先进产能占比。安全生产标准化矿井动态达标40座以上，其中一级矿井15座以上，所有生产矿井要达到二级以上水平。完成13座煤矿核增产能任务，新增先进产能2500万吨。全年生产原煤1.6亿吨，生产洗煤2亿吨。发挥晋北到江苏、蒙西到天津南两条特高压通道优势，借助苏晋能源落户，扩大电力外送规模，提高电厂机组利用小时，完成发电量430亿度。加强煤炭行业管理，加大超能力生产和超层越界开采整治力度，治理跑冒滴漏。煤电企业要坚持效益优先，与朔州发展同呼吸、共命运，履行社会责任，治理生态环境。

　　围绕“促新”，发展壮大特色产业。高端陶瓷和新材料产业，要以建设中国北方瓷都为目标，重点解决同质竞争、规模不大、品牌不响、附加值不高、配套不足等问题。落实扶持政策，支持技术创新，开发高科技工业瓷、高品位美术瓷、高品质建筑瓷。加快质量标准体系建设，创建朔州陶瓷品牌。要加强对外合作，通过兼并重组，壮大规模。发展配套产业，促进专业化分工协作，打造陶瓷产业集群。落实国发42号文件精神，争取国家发改委、工信部、科技部和科研院所支持，加快煤矸石、粉煤灰综合利用等新材料产业发展。新能源产业，要加快晋北风电基地建设，推动“光伏领跑基地”及“平价上网示范基地”申报，年内新增新能源装机50万千瓦。现代煤化工产业，发展煤制油、煤制气、煤制烯烃、煤制乙二醇产业，实现资源最大化、最高值化利用。晋北(朔州)现代煤化工基地要抓紧推进规划环评，抓住国家能源战略项目布局的有利契机，力争40亿立方米/年煤制天然气项目落地，积极争取400万吨煤制油项目，力争用3—5年时间把现代煤化工打造成为我市一大支柱产业。生物医药产业要重点推进怀仁医药园区建设，突出专业化，以园招商、以商招商，承接产业转移，做大产业规模，打造成为全市转型发展的新引擎。文化旅游产业，举右玉龙头，走生态之路，打长城品牌，借助《右玉和她的县委书记们》在央视热播产生的巨大影响，挖掘生态文化、历史文化内涵，讲好右玉故事，引入全国、全省具有较大影响的文体赛事，举办好森林音乐会、西口风情生态文化旅游节、右玉生态国际马拉松比赛、国际自行车职业联赛等活动，让右玉全域旅游迅速热起来、火起来，成为全市乃至全省文化旅游产业发展的一张名片，带动全市文化旅游发展。加快长城旅游公路建设。发展工业旅游，筹划建设能源博物馆。城区神头海、怀仁金沙滩、山阴广武城、应县释迦塔、右玉杀虎口等景区景点，要引进战略投资，大力度开发建设。要加强与大同、忻州旅游同构，实现游览在周边、消费在朔州，让文化旅游产业成为“活力朔州”的重要支撑。草牧业与农产品深加工产业，要发挥我市草食畜和杂粮生产黄金产业带优势，扩大优质牧草和富硒富锌优质杂粮种植，提升标准化养殖规模，重点发展以杂粮、油料和草食畜为主的农产品加工业。商贸物流产业，重点发展煤炭物流。我市是西煤东运、北煤南运的重要节点，是大秦线货运的源头，是准池、准朔、朔黄、神朔铁路线交汇地，也是连接大运、荣乌两条高速公路的重要枢纽，境内铁路总里程612公里，煤炭铁路运量全国最大，发展煤炭物流极具优势。要抓住公转铁契机，以内陆港为载体，吸引过境的陕蒙周边资源，推动煤炭生产由资源开发型向物流储配基地转变，由单一的生产型向生产+物流型转变，实现为终端用户定制化生产。搭建由供应商、需求方、贸易商和服务商多方参与的公共信息服务平台，开发集装箱多式联运，拓展煤炭物流增值性服务，同时要利用公铁联运，打通大宗商品供应链的上中下游，融入“一带一路”，探索开行国际联运班列，打造连通晋陕蒙、辐射京津冀的煤炭物流超市和返程物流集散新平台。

　　围绕“保障”，激活发展动力。提升金融服务能力。金融是朔州发展的动力源，也是短板。要进一步扩大投放总量，提升投放水平。重点解决金融机构不敢贷、不愿贷、不能贷问题。金融机构要认真落实中共中央办公厅、国务院办公厅《关于加强金融服务民营企业的若干意见》，切实履行服务民营企业第一责任人职责，年内全市银行业机构存贷比力争提升5个百分点。六大国有银行既要重煤电，更要重小微。要进一步创新融资模式，分区域、分行业、分企业设计产品，满足中小企业多样化融资需求。完善银行包联企业工作机制，所有银行要深入研究产业、企业、市场，提高授信放贷能力。对生产经营正常的企业不得随意抽贷断贷。壮大地方性银行业机构实力，完成右玉县农信社改制，启动应县农信社改制并力争在年内实现不良资产出表。要强化政府增信体系建设。壮大市县政策性融资担保机构实力，吸引国有、民营资本参股，市级担保机构资本金规模力争达到10亿元;各县(市、区)政策性担保机构资本金达到5000万元。完善银行业机构支持地方经济发展考核评价体系，运用财政资金和行政事业单位对公存款，有效引导和撬动银行信贷投放。要提升企业融资能力。抓好企业不动产确权登记，年内全部解决规上工业企业历史遗留的产权不明晰、资产不完整问题，朔州经济开发区、怀仁市要率先突破，上半年取得实效。中小企业要加强诚信体系建设，提升公司治理能力，为金融支持提供必要基础条件。要拓宽企业融资渠道。利用多层次资本市场助力中小企业融资，加大中小企业通过“新三板”、股权交易中心融资的扶持和引导。推动玉龙化工通过资产重组上市。加快市政银城市建设投资公司市场化运作，提升投资建设能力。强化科技和人才支撑。在新材料、固废利用、煤化工等领域，组织攻关一批重大科技项目，转化一批科技成果。引导企业加大科研投入，支持优势企业设立院士工作站、博士后工作站、重点实验室和工程技术研究中心，建设中试基地。新培育10家高新技术企业。加强与省农科院合作，推进朔州分院建设。设立人才奖励基金，筹建人才公寓，建立人才购房租房货币补贴制度，引进百名高层次人才。实施“人才回流计划”，大力度吸引朔州籍科学家、企业家，关注家乡、服务家乡、建设家乡。加快知识型、技能型、创新型人才队伍建设，培养千名本地专业技术人才，让各类人才尽展其能、创新活力竞相迸发。

　　各位代表，稳煤促新、转型发展是资源型城市的唯一出路。实现转型发展，提升发展质量，抓好基础性、支撑性要素尤为关键。我们就是要立足煤电优势，着力接环补链，推进提档升级，聚合金融、科技、人才等各类要素，重点发展煤化工、煤炭物流、高端陶瓷、文化旅游等产业，为转型发展、高质量发展注入新动能。

　　(三)全力推进重点领域改革，增强经济社会发展活力

　　推动县域经济差异化发展。加快制定差异化考核指标体系。各县(市、区)要立足资源、区位、产业优势，围绕如何放大优势、凸显优势，在精准招商、旅游提质、煤电延伸、陶瓷提档、新能源扩规等方面，谋划推动一批重点项目。朔城区要大力发展现代服务业和城郊型农业，提升公共服务水平，增强市区辐射力、带动力。平鲁区要聚焦煤电，发展煤炭物流，延伸产业链条，打造综合能源基地。怀仁市要突出医药、陶瓷、肉羊深加工特色，提档升级、做大做强，招商引资额要占到全市一半以上。山阴县要加快在现代煤化工产业上突破，在乳制品产业上领跑全市、全省。应县要发展好现代农牧业，扩张提升陶瓷产业，做足做好木塔文章。右玉县要举好生态文化旅游龙头，加快高质量发展三年行动计划，努力打造全国知名生态文化旅游目的地、践行“两山”理论示范区和全域旅游样板区。

　　推动开发区改革发展。市县要支持开发区深化“三化三制”改革，推进开发区行政管理体制和市场化运营模式分离改革，促进开发区治理体系和治理能力现代化。复制推广转型综改示范区改革创新经验，落实对开发区授权，努力做到办事不出区。鼓励市场主体参与开发区建设。强化对开发区项目建设的考核，年内朔州经济开发区要落地3—5个10亿元以上项目，其他开发区要落地2—3个5亿元以上项目。按照国家级开发区标准，全力推动朔州经济开发区建设。

　　破解民营经济发展难题。落实支持民营经济发展48条措施。市财政设立1亿元发展资金，用于生态环境建设、企业技术改造、科技人才发展。培育不少于32家“小升规”企业。保护民营企业合法权益，清理拖欠民营企业账款。强化企业家队伍建设，培训3000名企业管理人员。

　　创新用地机制。用足用活土地政策，积极推进城乡建设用地增减挂钩、农村闲置农房和宅基地盘活利用、工矿废弃地复垦，加大造地力度，保障开发区和省市重点项目用地需求。完善农村集体经营性建设用地产权制度。加快推进“房地一体”的农村集体建设用地和宅基地使用权确权登记颁证。推广右玉县闲置凋敝宅基地整治盘活利用试点做法。加大高标准农田建设力度，增加粮食产能储备，释放结余指标。抓住有效“窗口期”，年内各县(市、区)完成2000亩增减挂钩任务，储备充足的耕地占补平衡指标，实现保护耕地与保障发展双赢。有序推动社会资本参与土地整理项目，实现土地整理投入多元化、实施模式多样化，提升土地整理水平。

　　推动电力体制改革。“窝电”难题和“售电”瓶颈是制约我市电力工业发展的最大问题。支持苏晋能源将更多电源点纳入统购统销范围。鼓励煤电企业复制苏晋能源模式，通过资产整合、股权重组，推进煤电一体化。推进电力直接交易，积极培育售电公司，推动山阴北周庄工业园区增量配电改革迈出实质性步伐。落实园区长协交易协议，交易电量5亿度，为非煤企业节约用电成本1亿元左右。年内再新增长协交易电量5亿度，让发电企业增电量，让用电企业降成本，使低成本电价转化为实体经济的竞争优势，成为招商引资的重要筹码。

　　推动财税改革。推进财政预算绩效管理改革，各级财政压缩一般性支出，盘活财政存量资金，提高财政资金使用效率。落实国家降费减税政策。积极争取专项政府债券支持。推进个人所得税、增值税改革，做好社会保险费和非税收入承接。

　　深化地企合作。中煤驻朔企业是我市能源基地建设的重头，中煤平朔将全方位、大力度支持朔州发展，深度参与大原高铁、煤化工、煤炭物流和城市建设。要进一步加强与中煤平朔、同煤集团、中电神头、太原铁路、国网供电、金融机构等合作，推动地企互利双赢、共同发展。

　　(四)全力打造对外开放新高地，提升对外开放水平

　　构建区域协同发展新格局。发挥中心城市对宁武、神池及河保偏地区的辐射带动作用，主动融入太原都市圈。加强与大同市合作，实现资源同享、生态环境共建共享、旅游同构、基础设施互联互通。对接呼包鄂榆城市群国家战略走廊，实现与陕北、蒙东地区联动发展，加强能源开发利用、特色旅游、绿色农牧产品加工等方面的合作。加强与京津冀地区、雄安新区在产业、人才、科技、文化方面的合作，与秦皇岛港、黄骅港合作，构建内陆地区与沿海港口的大通关平台。

　　加大招商引资力度。抓住北京非首都功能疏解机遇，加强与北京跨区域产业协作中心合作，承接产业转移。组织长三角、晋商晋才家乡行以及央企、知名民企、跨国公司入朔等招商活动，开展日韩、美加、北欧等境外招商推介。组织参加中国国际进口博览会。加强招商项目跟踪，确保项目落地。落实招商引资政策，让来朔投资者盈利，把“获利朔州”叫响，推成招商引资名片。

　　着力构建开放平台。筹备召开首届能源大会。继续办好塞上长城国际旅游节、以陶瓷为主的工业品交易会、以羔羊肉为主的特色农产品交易会、亚洲粉煤灰大会“一节三会”。打造“N节N会”招商平台，每个县(市、区)至少举办两场节庆会展。

　　支持外向型企业发展。支持和激励晋利糖果、尊屹陶瓷、右玉图远、鑫霏农业、晋坤矿产品、三元炭素等外贸型企业扩大出口份额，开拓新兴市场。朔州海关要加强对外贸型企业的业务指导，提升通关效率，降费减负，让外贸企业享受到实实在在的“利好”。

　　各位代表，朔州是改革开放的试验田，现在已进入改革深水区。传承改革开放基因，打造改革开放高地，推动改革开放再出发，是朔州当前和今后发展的关键一招和必由之路。我们就是要立足本地实际，发挥土地广阔、电力充沛、县域发展各具特色的现实优势和对外开放的潜在优势，全力推进市场化改革，构建全方位开放格局，最大限度释放改革开放红利，让改革开放不断成为朔州发展的强劲动力。

　　(五)全力实施乡村振兴重点工作，巩固发展“三农”持续向好形势

　　实施朔州苜蓿行动。加快建设全国草牧业示范市、雁门关农牧交错带核心区，提高牧草质量标准，建设牧草质量检测化验室，实施优质牧草替代进口战略，推进朔城区、山阴县、怀仁市3个5万亩苜蓿基地建设，实施20万吨苜蓿青贮行动，建设5万平方米标准化干草棚、10万立方米青贮窖、2个万吨牧草储备库，把草业发展成为我市农业的一大新兴产业，建成晋蒙地区优质牧草种植基地。

　　强化农产品品牌创建。打造“质量强市”，推动农业标准化生产，加快农业生产技术操作规程和农产品质量标准制定，叫响“右玉羊肉”“平鲁红山荞麦”“怀仁绿豆”“应州蔬菜”“山阴乳制品”“城区蔬菜水果”等一批农产品品牌，认证绿色和有机农产品15个、农产品地理标志1个，农业标准化生产率达到38%以上。

　　提升农业现代化经营水平。调整“粮经饲”种植结构。坚持农牧循环、三产融合，用工业化推进农业发展，促进农旅深度融合。发展有机旱作农业，抓好全省示范县山阴县和示范片怀仁绿豆、平鲁红山荞麦片区建设。推动规模健康养殖，新建改建20个奶牛园区，建设3个5万平方米肥羔羊现代化养殖示范点。全市奶牛存栏19万头，羊饲养量630万只，生猪饲养量100万头。抓好非洲猪瘟防控。布局种、养、加以及市场、物流相配套的全产业链项目。龙头企业、农村专业合作社与农户建立紧密的利益联结机制，让农民分享产业链增值收益。加快农产品流通体系建设，实施“互联网+”农产品出村进城工程，鼓励龙头企业在沿海城市、区域中心城市建立展销中心，加大对农产品销售大王、名优农产品生产企业的奖补力度，打通我市农产品通往京津冀、长三角、珠三角的绿色通道，让好产品卖出好价钱。农产品加工业销售收入要达到233亿元，增长6.5%。

　　健全农业担保扶持机制。创新政银保联动机制，发挥农牧交错带产业发展基金作用，加强与省农担公司合作，重点强化30户农产品加工企业和30家养殖园区的金融支持。农商行、农村信用社新增贷款不低于30亿元。完善农业保险和大灾风险分散机制，推进玉米、奶牛、能繁母猪政策性保险全覆盖。开办杂粮、干鲜果、蔬菜、牧草、中药材等特色农作物险种。

　　决战决胜脱贫攻坚。把脱贫攻坚摆在乡村振兴的优先位置，坚持摘帽不摘责任、不摘政策、不摘帮扶、不摘监管，巩固提升贫困村和边缘人口较多的重点村，防止贫困户返贫、低收入非贫困户致贫。推行保险防贫，将6.5万建档立卡贫困人口和低收入非贫困人口全部纳入防贫保险范围。巩固提升贫困村和重点村人居环境。加大产业扶贫力度，强化龙头企业带动，激发群众内生动力。

　　深化农村改革。落实农村土地承包期再延长30年政策。推进承包地“三权分置”改革，引导农村土地经营权向种田能手和新型经营主体有序流转，推动适度规模经营。6月底完成农村集体资产清产核资，年底完成40%以上的农村集体产权制度改革。稳慎推动农村宅基地制度改革。加快市、县、乡农村产权交易市场建设运行，激活农村生产要素，增加农民财产性收入。

　　改善乡村人居环境。落实好农村人居环境三年整治行动，百村示范、千村整治，加大拆违治乱、生活垃圾治理、污水治理、厕所革命、卫生乡村建设力度。生活垃圾有效治理率达到78.5%。推动桑干河沿线建制镇生活污水处理设施建设。新创建60个美丽乡村，打造30个乡村振兴示范村。改厕4.3万个，卫生厕所覆盖率达到45%。坚决打赢“大棚房”问题专项整治攻坚战。

　　(六)全力提升城市品质，打造宜居宜业幸福区

　　编制国土空间规划。制定《美丽朔州2050年空间发展战略规划》，推进市县《国土空间规划(2035)》编制，因地制宜开展乡镇国土空间规划、村庄规划编制，完善市县国土空间规划信息平台和空间规划一张图，开展资源环境承载力和国土空间开发适宜性双评价工作。

　　完善城市功能。统筹推进城市基础设施和公共服务设施建设，加快道路、桥梁、供排水、污水垃圾处理、供热供气、商业网点等建设，提升城市品质，推动城市发展向内涵提升转变。推进总投资11.7亿元的七里河六座桥及连接线建设。加快建设市公交调度中心，改建100个公交候车亭，开通两条公交线路，实现市区公交线路全覆盖。

　　加强城市管理。按照统规、共建、统管、共享的思路，理顺城市一体化管理体制。加大城市管理综合执法力度。完善数字城管系统，推进智慧城市建设。深化“六城联创”，巩固提升国家园林城市、全国双拥模范城创建成果，加快国家卫生城市、国家环保模范城市、国家文明城市、国家食品安全示范城市创建步伐。

　　(七)全力推进生态文明建设“六大工程”，进一步巩固提升生态优势

　　大力开展植树造林。加快市域内管涔山生态防护林、洪涛山劣质地造林、恒山水源涵养林建设，加快外长城沿线生态旅游带、城郊森林景观带、桑干河生态修复带、南坡区生态经济林带建设，提升平原区绿化水平。完成营造林38.47万亩。怀仁市实现全域绿化。

　　坚决打赢蓝天保卫战。持续推进治污、控煤、管车、降尘。实施城中村、城边村集中供热改造，市区清洁取暖覆盖率达到100%;县(市、区)建成区完成16000户清洁取暖改造任务，覆盖率达到80%。加强农村洁净型煤供应，清洁取暖覆盖率力争达到50%以上。加快燃煤锅炉改造。加大扬尘污染治理，全面推进建筑工地绿色施工，继续推进柴油货车和散装物料运输车污染治理。

　　持续打好碧水保卫战。抓好化工、陶瓷、医药、屠宰企业生产废水达标排放改造，完成市县污水处理厂提标改造。持续推进桑干河清河行动。落实河长制，全面加强入河排污口监管，确保国、省考断面达标。

　　统筹打好净土保卫战。制定实施土壤污染防治行动计划。开展工业企业用地和农用地土壤污染详查。加强采煤沉陷区、煤矸石、粉煤灰治理，防范土壤环境风险。积极申报建设“无废城市”试点。对中央、省环保督察交办和反馈问题无条件整改到位。

　　强化环保倒逼机制。围绕生态城市定位，在发展理念上严守生态红线，在目标取向上改善生态民生，在产业选择上突出生态为基，更加自觉地贯彻绿色发展理念。强化环境容量管理，推动产业绿色发展，实现经济发展与环境保护协同共赢。

　　(八)全力抓好民生事业，增强人民群众获得感幸福感

　　优先发展教育事业。推动城乡义务教育一体化发展，启动县域义务教育优质均衡创建工作。振兴乡村教育，做好乡村学校布局优化和资源整合。规范民办教育管理，大力发展职业技术教育。抓好学前教育，认定普惠性幼儿园76所，毛入园率提高3个百分点。发展特殊教育，保证残疾儿童入学率达到96%。

　　促进就业创业。鼓励以创业扩大就业。继续实施全民技能提升工程，完成对5万农民的技能培训。加强就业服务，打造就业公共服务平台。加强高校毕业生、建档立卡贫困人口、特困人员、军队退役人员、零就业家庭就业工作。开展免费法律咨询便民服务。

　　加强社会保障。提高城乡居民最低生活保障标准，每人每月提高30元。提高特困供养人员标准，每人每年达到5500元。完善城乡居民大病保险制度，把更多的好药纳入医保。加快养老服务业发展，构建医养结合养老模式，推进农村老年人日间照料中心建设，市社会福利院老年公寓投入使用。加强住房保障，棚户区改造开工2514套，基本完成建成区城市棚户区改造任务;改造农村危房4117户，实现建档立卡贫困户、低保户等四类重点对象危房“静态清零”。实施经济困难的高龄和失能老年人关爱行动工程。继续为全市贫困大学生每人发放助学金2000元。

　　提升全民健康水平。深化公立医院综合改革，建立现代医院制度。深化县乡医疗卫生机构一体化改革，加大优质资源下沉力度，推开医联体建设，全面落实分级诊疗制度。继续实施“五个一”兴医工程，建设1个人口健康信息网、打造10个领军临床专科、完善市级10大卫生健康基础工程、扶持100个基层医疗卫生单位、招录1000名全日制医学生。启动朔州中医院项目前期。将国定贫困县农村妇女免费“两癌”检查服务扩大到全部贫困县，为怀孕妇女提供免费产前筛查与诊断服务，实施残疾预防重点干预和残疾儿童抢救性康复项目。

　　提升公共文化服务水平。推动“城市书屋”建设，打造“书香朔州”。提升乡村公共文化服务设施建设水平。实施“送戏、送电影、送图书”下乡活动。启动朔州大剧院前期工作。组织好二青会击剑、马术比赛。开展庆祝新中国成立70周年、建市30周年等活动。

　　(九)全力维护社会和谐稳定，提升人民群众安全感

　　积极稳妥防范化解重大风险。落实省委关于坚决打好防范化解重大风险攻坚战《方案》，防范化解政治、社会、生态环保等八大领域风险。规范政府举债融资机制，强化债务风险防控。严厉打击非法集资活动，打击恶意逃废金融企业债务行为，维护金融秩序和金融安全。

　　坚持不懈抓好安全生产。压实“党政同责、一岗双责、企业(单位)主责、失职追责”责任，继续实施安全生产最差考评管理，推行“隐患排查治理清单”制度和专家例行会诊制度，切实加强隐患排查治理。抓好煤矿、道路交通、人员密集场所、危化品等重点领域的安全工作，促进安全生产形势持续好转。

　　加强和创新社会治理。完善社会治安综合治理长效机制。加强食品药品安全监管，严防环保突发事件，严控群体性事件，做实做细各类利益诉求群体的信访维稳工作，有效化解各类社会矛盾，维护社会和谐稳定。完成总投资2.7亿元的全市域“雪亮工程”建设。持续推进“扫黑除恶”专项斗争，不断提升人民群众安全感。

　　支持国防动员和后备力量建设，深入推进军民融合发展，加强双拥和退役军人服务管理工作。支持工会、共青团、妇联等群团组织开展工作，发展妇女、儿童、老龄、慈善和红十字会等事业，加强气象、地震、人防、档案等工作，做好民族宗教、外事、侨务、对台等工作，为我市改革发展凝聚强大合力!

　　三、全面加强政府自身建设

　　坚持以政治建设为统领，自觉用习近平新时代中国特色社会主义思想武装头脑、指导实践、推动工作，树牢“四个意识”，坚定“四个自信”，做到“两个维护”。深入开展“不忘初心、牢记使命”主题教育，扎实开展“改革创新、奋发有为”大讨论，聚焦“六个破除”，推动思想再解放、改革再深入、创新再发力、开放再提质、工作再抓实，持续加强“三基建设”，建设人民满意的服务型政府。

　　打造“六最”营商环境。做好市级机构改革转隶组建、“三定”规定，完成县(市、区)机构改革，优化职能配置。推行“互联网+政务服务”，建成联通省级、覆盖全市的一体化政务服务平台。持续推进审批服务便民化，推动政务服务事项“马上办、网上办、就近办、一次办”，网上办理达到80%以上。全面推行企业投资项目承诺制，推动“多审合一”改革，缩短审批时间。深化商事制度改革，全面推开“证照分离”改革，推进“照后减证”，建立跨部门、跨领域“双随机、一公开”监管执法机制。

　　提升政府服务水平。加强研究，准确把握国家和省宏观政策，继续谋划推动牵引性、基础性、全局性重点工作、重大项目和重要改革，着力破解发展瓶颈。加强经济运行研判，精准调控、精细管理、高效服务，提升经济运行质量和效益。开展政企互动政策解读对接交流活动，创造性地贯彻落实党中央、国务院大政方针和省委、省政府决策部署，提高各级干部和企业家运用政策、推动发展的能力。组织干部对口省直部门双向挂职，提高工作水平，更加积极争取上级政策和项目支持。

　　全面推进依法行政。自觉运用法治思维和法治方式推进工作，严格执行人大及其常委会决议决定，认真办理人大代表建议、政协委员提案，主动接受人大、政协监督。做好规范性文件备案审查工作。发挥好政府法律顾问作用。坚持依法决策，完善重大行政决策公众参与、专家论证、合法性审查等制度。做好行政复议和行政应诉工作。加强审计监督，全面推行内审制度。

　　担当作为狠抓落实。各级领导干部要大力弘扬右玉精神，带头解放思想，带头改革创新，始终保持攻坚克难、奋发有为的精神状态。主要负责同志要当好“施工队长”，立说立行、真抓实干，矛盾面前不回避、困难面前不推诿，政策在一线落实，工作在一线推进，问题在一线解决，在推动高质量发展中干出精气神、干出新业绩。

　　加强政府廉政建设。深入推进政府系统党风廉政建设和反腐败斗争，贯彻执行中央八项规定及其实施细则精神，持之以恒纠正“四风”。牢固树立“过紧日子”思想，厉行勤俭节约，反对铺张浪费，把有限的资金用在改善民生、推动发展上。坚决破除形式主义、官僚主义，整治庸政懒政怠政、不作为慢作为乱作为现象，努力营造风清气正的发展环境。

　　各位代表，我们面临的任务艰巨繁重，肩负的使命重大光荣。让我们更加紧密地团结在以习近平同志为核心的党中央周围，在市委的坚强领导下，改革创新、奋发有为，凝心聚力、真抓实干，奋力建设塞上绿洲、美丽朔州，以优异成绩迎接中华人民共和国成立70周年!
